# Alkyl and Aromatic Amines as Digestive Ripening/Size Focusing Agents for Gold Nanoparticles

**DOI:** 10.3390/nano3030370

**Published:** 2013-07-05

**Authors:** Yijun Sun, Deepa Jose, Christopher Sorensen, Kenneth J. Klabunde

**Affiliations:** 1 Department of Chemistry, Kansas State University, Manhattan, KS 66506, USA; E-Mails: julia.yj.sun@gmail.com (Y.S.); djose@ksu.edu (D.J.); 2 Department of Physics, Kansas State University, Manhattan, KS 66506, USA; E-Mail: sor@phys.ksu.edu

**Keywords:** amines, gold nanoparticles, inverse micelle, metal vaporization

## Abstract

Both long chain alkyl thiols and alkyl amines behave as size focusing agents for gold nanoparticles, a process that is under thermodynamic control. However, amines do not oxidize surface gold atoms while thiols do oxidize surface gold to gold(I) with evolution of hydrogen gas. Therefore, alkyl amines participate in digestive ripening by a different mechanism. The efficiency of alkyl amines for this process is described and compared, and ultimate gold particle size differences are discussed. Reported herein is a detailed investigation of alkyl chain lengths for alkyl amines, aromatic amines (aniline), and unusually reactive amines (2-phenylethyl amine). Also, two methods of preparation of the crude gold nanoparticles were employed: gold ion reduction/inverse micelle *vs.* metal vaporization (Solvated Metal Atom Dispersion—SMAD).

## 1. Introduction

The fascinating and unique behavior of ligand capped nanoparticles toward certain preferred sizes has been observed by many workers. Size and monodispersity can be affected by a thermodynamic process called digestive ripening, which can be controlled by ligand choice, solvent choice, and temperature [1,2,3,4,5,6]. However, there have been many reports concerning theoretical approaches [7], a wide variety of synthetic approaches utilizing kinetic control and different media, and how size and properties are affected [8,9,10,11,12,13,14,15,16,17,18,19,20,21,22,23,24,25,26,27,28,29,30,31,32,33,34,35,36]. Also, several reviews have been published [37,38,39]. Alkyl amines, alcohols, phosphines, silanes, halides, and thiols have been compared as ligands for digestive ripening [1,2,34,38,40,41,42] and thiols are the most effective at yielding monodisperse gold, but amines work well too, yielding nanoparticles somewhat larger, but reasonably monodispersed.

Recently, we determined that hydrogen gas is evolved when thiols attack and ripen gold nanoparticles, and the hydrogen was provided by sacrifice of surface nanoparticle gold [43]. On the other hand, with alkyl amines as ligands, no hydrogen was evolved. Therefore, slightly different processes are involved with thiols *vs.* amines.

In order to carry out the hydrogen evolution measurements, it was necessary to exclude complex mixtures and large volumes of solvents, and so a metal vaporization method was employed to prepare the gold nanoparticles, followed by ligand addition and hydrogen measurements.

However, in our earlier work where different ligands were compared, gold ion reduction/inverse micelle preparation methods were employed, and the ion reduction/inverse micelle method yields crude nanoparticles that are different from the metal vaporization (SMAD) method [4,44]. Therefore, it became necessary to carry out SMAD preparations of alkyl amine-gold systems, which are reported herein. Also, reported are comparisons of aromatic amines with aliphatic amines, unusually reactive amines, such as aniline and 2-phenylethyl amine, and a detailed comparison of alkyl chain length effects, and comparisons of the ion reduction/inverse micelle preparation procedure and the metal vaporization SMAD procedure.

## 2. Experimental Methods

### 2.1. Materials

Didodecyldimethylammonium bromide (DDAB) was purchased from Fluka and used as received. Sodium borohydride, gold chloride (99.99%), bulk gold, butylamine (98%), octylamine (98%), dodecylamine (98%), hexadecylamine (98%), octadecylamine (98%), aniline (98%), and 2-phenylethylamine (98%) were purchased from Sigma-Aldrich and used without further purification. Deionized distilled water was obtained from a Barnstead nanopure system. Toluene (99.9%), ethanol (99%) and Butanone (99%) were purchased from Fischer Scientific. The toluene was dried over sodium, and butanone was dried over K_2_CO_3_ and were distilled and degassed four times by the freeze-pump-thaw procedure before use in SMAD experiments.

### 2.2. Preparation of Au-alkylamine Colloids by the Ion Reduction/Inverse Micelle Method. Specific Methods for Gold Nanoparticles

#### 2.2.1. Preparation of the Crude Gold Colloid

The gold-amine as prepared colloid was prepared at room temperature using a DDAB/water/toluene inverse micelle system. Much [41,42,43,44,45] has been done to obtain nanoparticles with a fairly narrow size distribution by carefully controlling the amount of water, surfactant, and the rate of reaction, as well as the reaction temperature. In order to study the effect of digestive ripening on the particle size distribution, a smaller amount of DDAB surfactant was used to create a polydisperse colloid [1]. A typical synthesis is as follows. A 0.025 M micelle solution was formed by adding 156 mg of DDAB (3.8 × 10^−4^ mol) in 15 mL toluene, and the toluene was degassed by bubbling with argon gas 2 h prior to use. Then 51 mg gold chloride (1.5 × 10^−4^ mol) was measured and dissolved in the micelle solution and sonicated for 15 min, to obtain a clear reddish brown colored solution. Meanwhile, a 9.4 M NaBH_4_ solution was formed by adding 0.178 g NaBH_4_ (4.7 × 10^−3^ mol) to 0.5 mL deionized water, 54 μL of that (5 × 10^−4^ mol) was added dropwise to the gold chloride micelle solution. The color of the solution changed to dark brown within one minute. Thus, the gold colloid was obtained after vigorously stirring for 30 min at room temperature. Then 5 mL of the gold colloid was transferred to a separate 30 mL vial to which a different amine was added at the bottom, together with a stirring bar. To keep the molar ratio of Au/amine about 1:30, phenethylamine (0.25 mL), butylamine (0.25 mL), octylamine (0.25 mL), dodecylamine (0.308 g), hexadecylamine (0.401 g), and octadecylamine (0.448 g) were used during these experiments. After stirring for 1 min within each vial, the color of the colloid turned to purple. Similar to Dodecanethiol, these amines also have an affinity to the gold surface [46] which results in a change of the interaction strength between the particles. The purple color was caused by the aggregation of the gold particles [47,48,49].

The amine capped gold nanoparticles were separated from the DDAB, excess amine, and the reaction by-product by precipitating with 15 mL of ethanol. After letting the vial stand undisturbed overnight, the particles, which settled down to the bottom, were isolated from the supernatant by decanting and vacuum drying.

#### 2.2.2. Digestive Ripening

**Figure 1:**
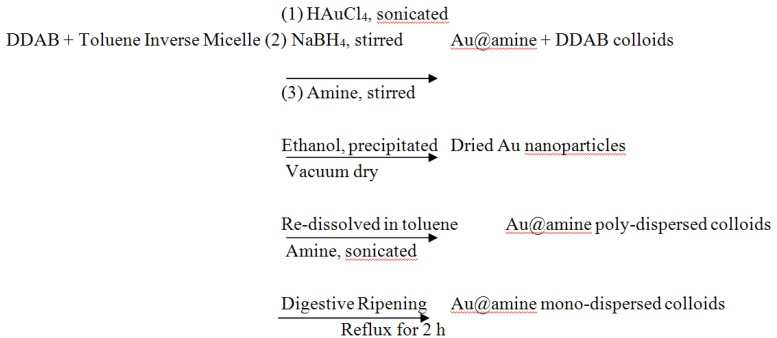
Schematic representation of the synthesis of gold colloid by inverse micelle method and digestive ripening process.

The dried precipitates were redissolved into 5 mL toluene and transferred to a 50 mL round bottom flask, and the same amount of amine was added to the flask for the digestive ripening process. Reflux of each mixture for 2 h under an argon atmosphere led to the formation of monodisperse colloids. Figure 1 shows the inverse micelle procedure and digestive ripening.

### 2.3. Preparation of Au-butylamine, Au-octylamine, Au-dodecylamine, Au-hexadecylamine, Au-octadecylamine as Prepared Colloids by SMAD Method

#### 2.3.1. Preparation of the Condensing Solvents

During the SMAD procedure, the distilled and degassed toluene and butanone were used as the condensing solvents. They were degassed by the freeze-pump-thaw procedure, during which the solvent was placed in a Schlenk tube and first flash-frozen by using liquid nitrogen (77 K), then a vacuum was applied, and the tube was sealed. Next, using a warm water bath to thaw the solvent, bubbles of gas formed and escaped. This procedure was repeated four times until the pressure inside of the tube remained constant. While we carried out this procedure, we found that the temperature of the liquid nitrogen was so low that occasionally the Schlenk tube would break during thawing. So in order to avoid this problem, we dipped the Schlenk tube into a Dewar full of ethanol, then poured liquid nitrogen to freeze the ethanol, because the melting point of ethanol is lower than the solvents. As the temperature goes down to freeze ethanol, it is low enough to make toluene and butanone frozen. This procedure proved to be a better way to avoid breaking Schlenk tubes.

#### 2.3.2. Preparation of the as Prepared Gold Colloid

The SMAD technique allows synthesis of pure gold colloids on a large scale. Earlier reports provide a detailed description of the Au nanoparticle preparation by the SMAD procedure [4]. The process was carried out under dynamic vacuum (4 × 10^−3^ Torr) in a sealed reactor immersed in a liquid nitrogen Dewar. Bulk Au (150 mg) was heated by the electrodes in the crucible and then evaporated and co-deposited with butanone vapors (120 mL liquid) on the walls of the reactor. At the end of this procedure, the liquid nitrogen Dewar was removed and the dark purple Au-butanone matrix was allowed to melt down. After stirring for about 1 h, the Au-butanone solution was siphoned into five vessels, which each contained a different amine (phenethylamine (0.20 mL), butylamine (0.20 mL), octylamine (0.20 mL), dodecylamine (0.224 g), hexadecylamine (0.292 g), and octadecylamine (0.325 g)) at the bottom. Each one was pumped down to evaporate butanone under vacuum. Then agitation was commenced for each vessel for about 2 h under argon atmosphere. Then the Au-amine colloid was diluted to 80 mL with toluene in preparation for the digestive ripening process.

#### 2.3.3. Digestive Ripening

**Figure 2:**
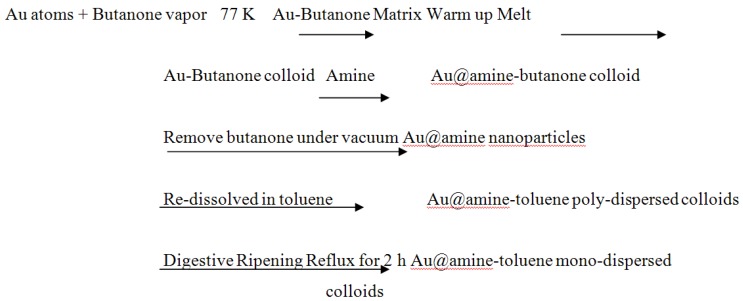
Schematic representation of the synthesis of gold colloid by Solvated Metal Atom Dispersion (SMAD) method and digestive ripening process.

The gold-amine-butanone colloid was then refluxed at the boiling point of toluene for 2 h under argon atmosphere. The final colloidal solution contains high quality Au nanoparticles stabilized by a different amine. Figure 2 shows the synthesis of the gold colloid procedure by the SMAD method and digestive ripening.

### 2.4. Characterization

#### 2.4.1. UV-Vis Spectroscopy

UV-Vis spectra were taken on a Spectral Instruments 400 series spectrophotometer (Varion Company, Walnut Creek, CA, USA) and toluene solvent was used.

#### 2.4.2. Transmission Electron Microscopy

TEM images were taken with a Philips EM100 microscope (FET Company, Hillsboro, OR, USA) operating at 100 kV. The particle size distributions were determined from a sample of a minimum of 200 particles. The diameter of these particles was measured by using software. Every distance was accurate to the fifth decimal place and the mean particle size and stand distribution were calculated based on these original data.

## 3. Results and Discussion

### 3.1. Gold-Amine by Inverse Micelle Method: The Effect of Alkyl Chain Length

A series of alkylthiols were used as capping ligands for gold colloids in our previous work [41,49]. It was very encouraging to find out that the digestive ripening process significantly reduced the average particle size and polydispersity, and formed separate particles with longer chain length (C_16_) thiols and aggregate into 3D superlattices with short chain length (C_8_ and C_10_) thiols. Those superlattices have similar solubility behaviors to those of normal molecular solids. Inspired by that finding, we have recently focused on using different amines as the capping ligand, by the same synthesis method, to investigate the particle behavior of the gold-amine colloids. The results are discussed in detail below.

**Figure 3:**
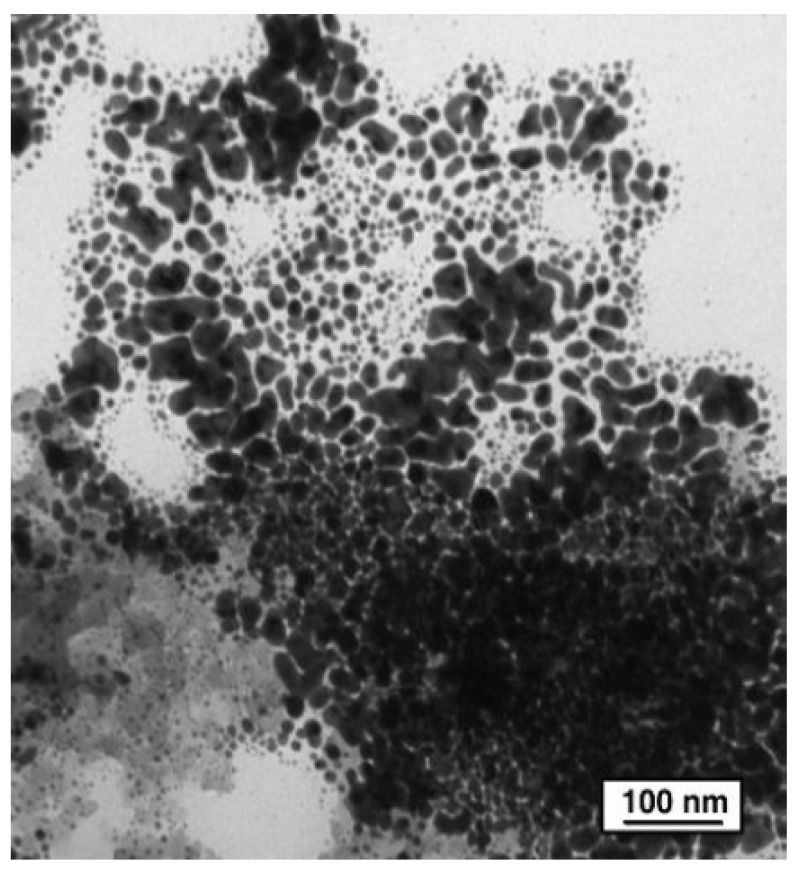
TEM images of Au-DDAB colloid in toluene by the inverse micelle method, before digestive ripening.

**Figure 4:**
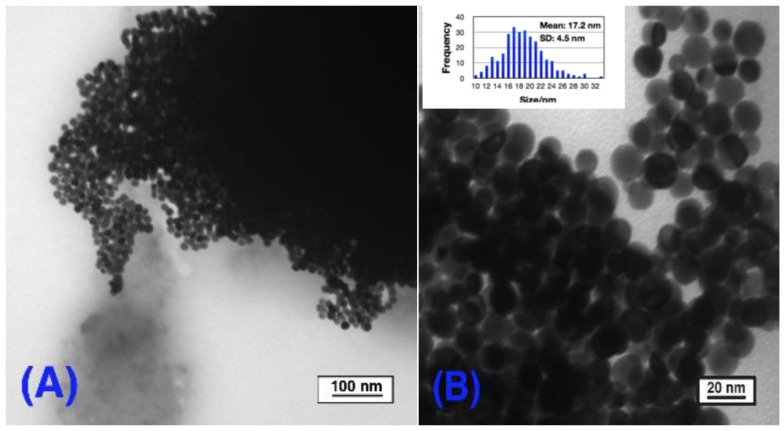
TEM images of (**A**) as prepared Au-butylamine colloid in toluene by inverse micelle method; (**B**) Au-butylamine colloid after digestive ripening 2 h in toluene.

**Figure 5:**
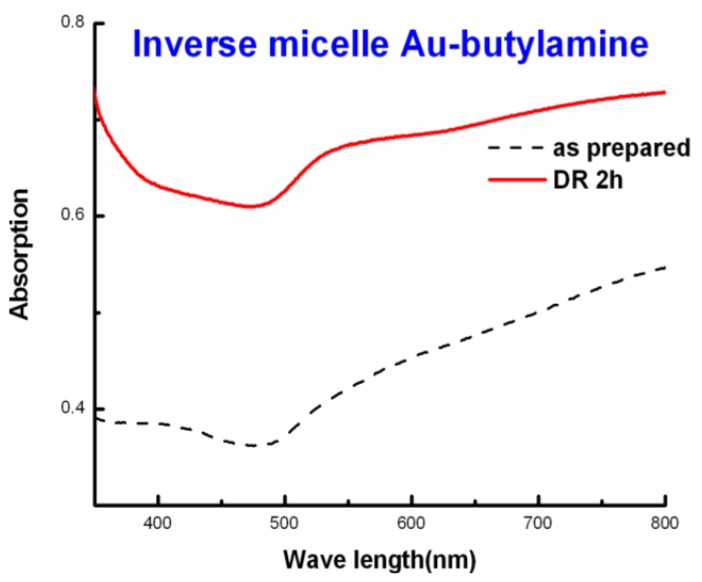
UV-Visible spectrum of Au-butylamine colloid by inverse micelle method before and after digestive ripening 2 h.

**Figure 6:**
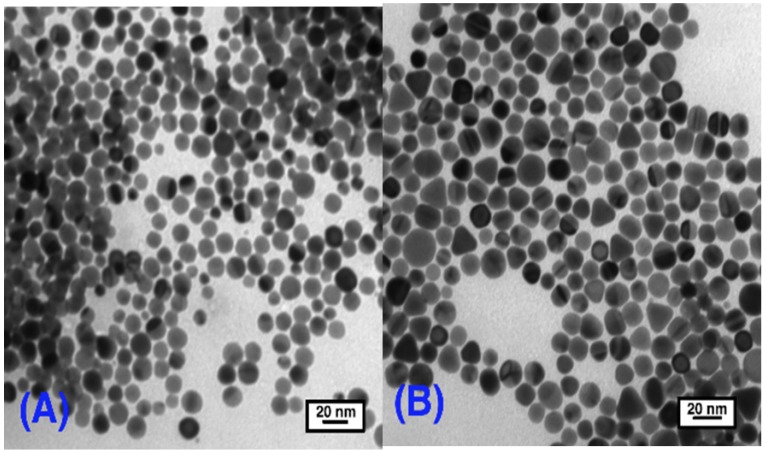
TEM images of (**A**) as prepared Au-octylamine colloid in toluene by inverse micelle method; and (**B**) Au-octylamine colloid after digestive ripening 2 h in toluene.

**Figure 7:**
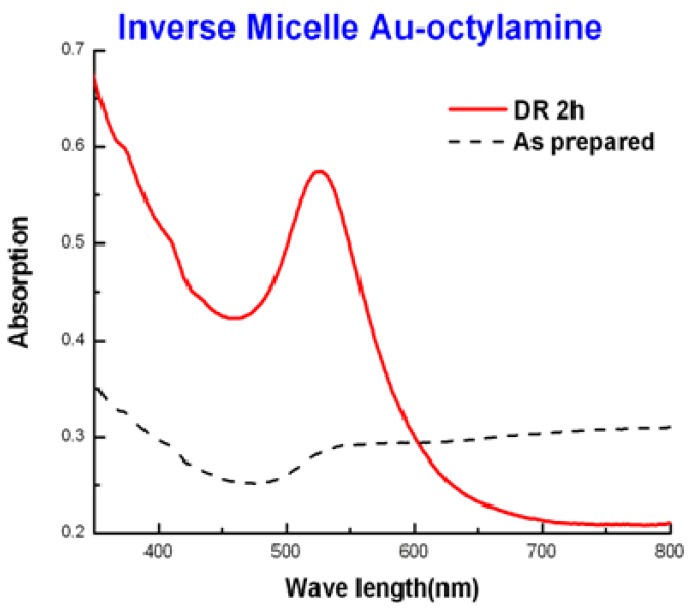
UV-Visible spectrum of Au-octylamine colloid by inverse micelle method before and after digestive ripening 2 h in toluene.

**Figure 8:**
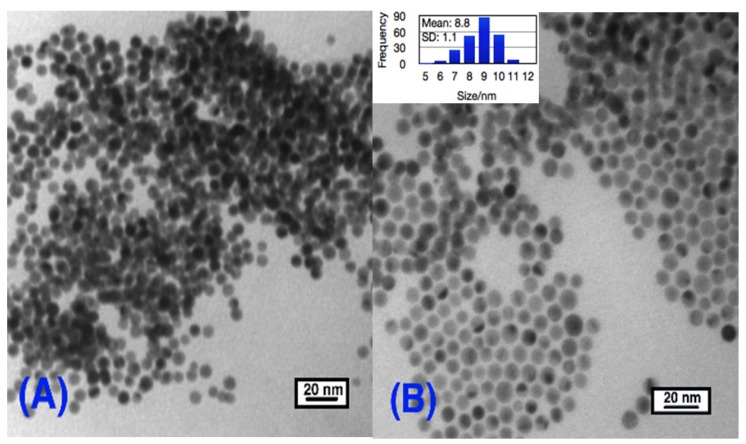
TEM images of (**A**) as prepared Au-dodecylamine colloid by inverse method in toluene; (**B**) Au-dodecylamine colloid after digestive ripening 2 h in toluene.

**Figure 9:**
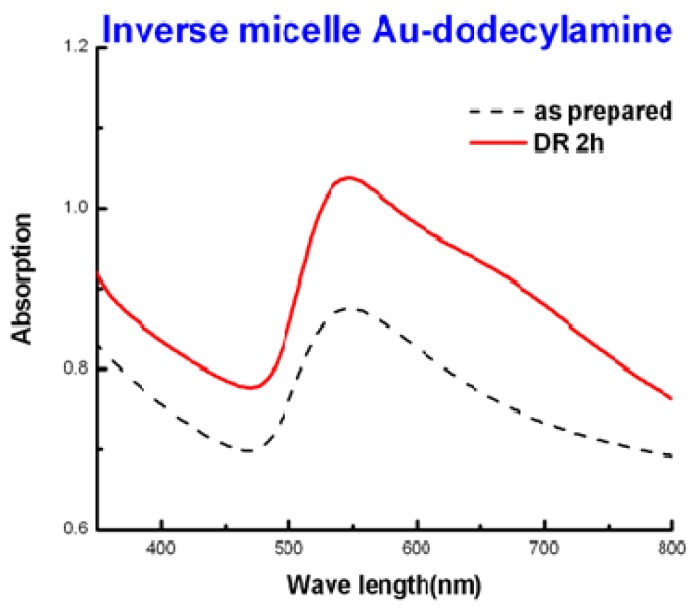
UV-Visible spectrum of Au-dodecylamine colloid by inverse micelle method before and after digestive ripening 2 h in toluene.

**Figure 10:**
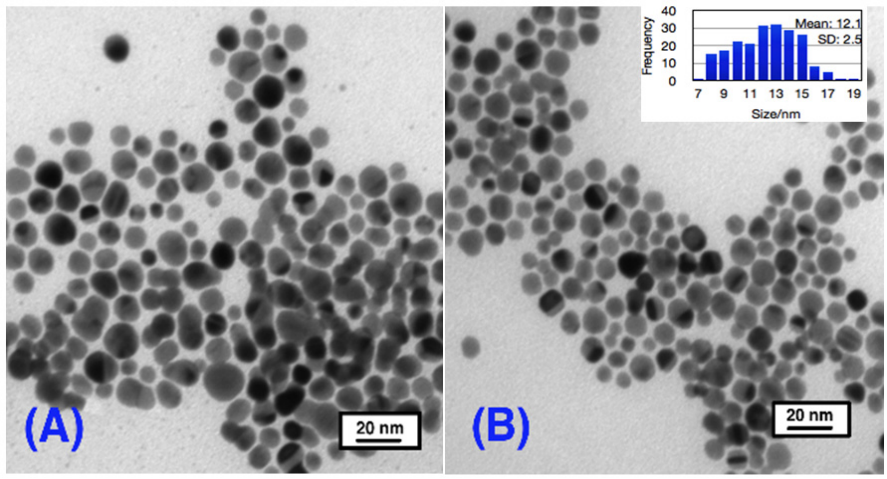
TEM images of (**A**) as prepared Au-hexadecylamine colloid by inverse micelle method in toluene; (**B**) Au-hexadecylamine colloid after digestive ripening 2 h in toluene.

**Figure 11:**
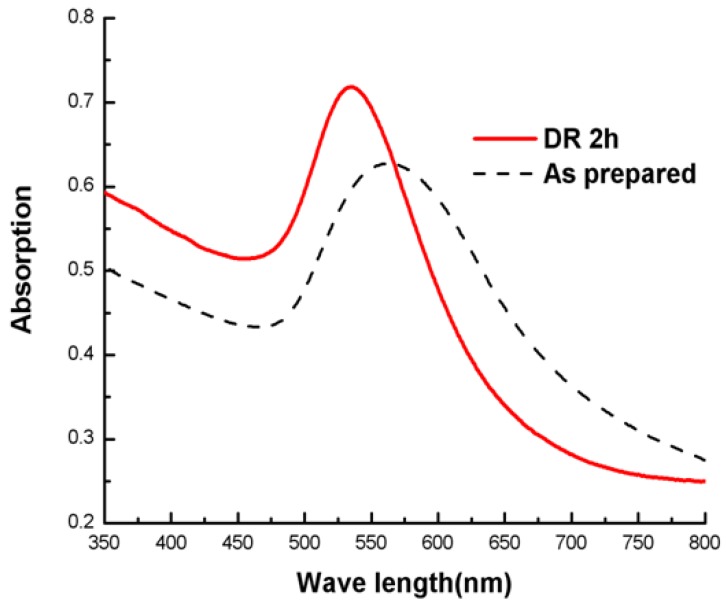
UV-Visible spectrum of Au-hexadecylamine colloid by inverse micelle method before and after digestive ripening 2 h in toluene.

**Figure 12:**
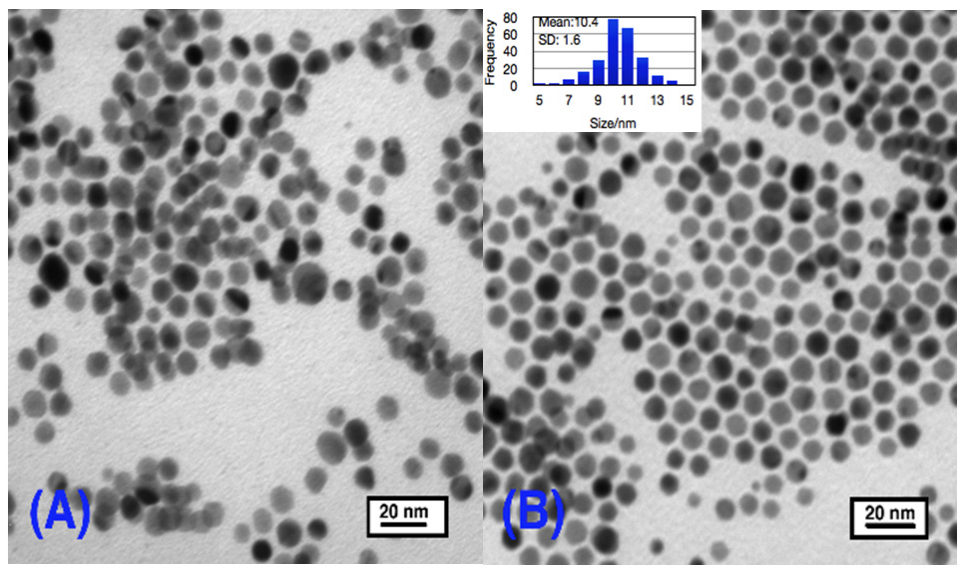
TEM images of (**A**) as prepared Au-octadecylamine colloid in toluene; (**B**) Au-octadecylamine colloid after digestive ripening 2 h in toluene.

**Figure 13:**
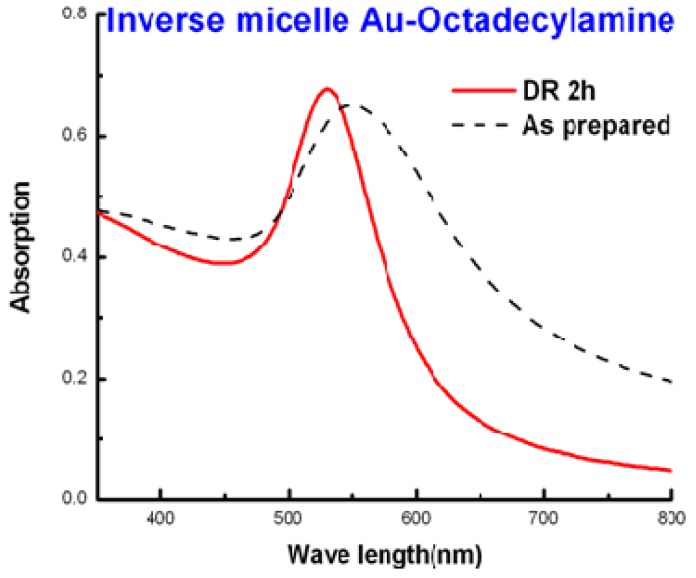
UV-Visible spectrum of Au-octadecylamine colloid before and after digestive ripening 2 h in toluene.

Different gold-amine colloids were synthesized by the inverse micelle method. The highly poly-dispersed gold colloids capped by DDAB are depicted in Figure 3. The addition of the different amines leads to many changes. Big polyhedral particles transformed into much more uniform particles, with sizes ranging from 8 to 22 nm. The morphologies and particle sizes of those gold colloids are very different due to the different capping ligands; they all easily settled down with different color, changing from dark blue to dark purple as the chain length of the ligands increases. The observations are: digestive ripening, during which all gold colloids were reddish purple homogenous solutions under reflux temperature of toluene, and visible changes appeared when they cooled down to room temperature. Au-butylamine (Au-C_4_N) colloids settled down quickly at the bottom, forming dark blue precipitates with almost clear supernatant. The dark purple particles of Au-octylamine (Au-C_8_N) colloid were also easy to precipitate, leaving a pink supernatant. The colloids of Au-dodecylamine (Au-C_12_N), Au-hexadecylamine (Au-C_16_N) and Au-octadecylamine (Au-C_18_N) are very similar to each other. After cooling down, the color changed from reddish purple to purple, some purple precipitates formed and the supernatant remained purple color. The morphologies and particle sizes of the gold colloids were characterized by TEM and UV/Vis (Figure 1, Figure 2, Figure 3, Figure 4, Figure 5, Figure 6, Figure 7, Figure 8, Figure 9, Figure 10, Figure 11, Figure 12, Figure 13) before and after digestive ripening. The digestive ripening procedure generally improved the particle size distribution. As can be seen from the TEM images (Figure 4, Figure 6, Figure 8, Figure 10, Figure 12), polydisperse colloids transformed into more monodisperse colloids that were more soluble. The mean diameters after digestive ripening of the Au-C_4_N, Au-C_8_N, Au-C_12_N, Au-C_16_N and Au-C_18_N colloids were 17.2 ± 4.5, 16.9 ± 2.8, 8.8 ± 1.1, 12.1 ± 2.5 and 10.4 ± 1.6 nm respectively. As for the morphology of these gold colloids, except for Au-C_4_N nanoparticles, which easily aggregated together and formed 3D superlattices, the others have a tendency to organize into 2D layers and the shape of the particles is more polyhedral rather than spherical after digestive ripening.

The UV/Vis absorption spectrum results (Figure 5, Figure 7, Figure 9, Figure 11, Figure 13) taken before and after digestive ripening of each gold-amine colloid are in complete agreement with the TEM observations. The optical spectra of the colloids of Au-C_4_N, Au-C_8_N and Au-C_12_N show similar broad plasmon absorption bands with no definite maximum. Au-C_16_N and Au-C_18_N colloid reveals a broad peak with a peak maximum around 570 nm. As for the digestive ripened gold colloids, all show a sharp and narrow peak around 530 nm, indicating a dramatic narrowing of the particle size and distribution, except for Au-C_4_N, which has a broad plasmon with a large tail above 700 nm.

It has been fully discussed now that the optical properties of gold colloids are mainly decided by the particle aggregation, particle sizes and distribution as well as interparticle separation [47]. Thus, the large tail observed for Au-C_4_N indicates that the particles are aggregating together which agrees with the TEM images (Figure 4). The red shift of the colloid in UV-Vis spectra was caused by the stronger electromagnetic coupling between the particles, which happens when the superlattices are bigger, and the ligand chain length separating the particles is smaller.

These observations suggest that the chain length of the amine has a great impact on the particle size variation and interparticle separation. Simple explanations like “lengthier ligands stabilize larger particles” [48,49] do not explain this phenomenon because as we can clearly see from the TEM images that C_12_N leads to the smallest particle sizes and narrowest size distribution (8.8 ± 1.1 nm), smaller than the longer ligands which are C_16_N (12.1 ± 2.5 nm) and C_18_N (10.4 ± 1.6 nm). Many proposals have been reported on the stabilization of nanoparticles suggesting that control may be due to thermodynamics other than the kinetics of nucleation and crystal growth [50]. These authors suggested that the nanoparticle size resulted from a combination of the ligand-gold binding energy and the surface free energy of the particles to reach the minimum energy of the whole system. The ligand-gold binding energy favors smaller particles with larger surface areas, while the surface free energy favors larger particles with smaller surface curvature. Therefore, these two effects oppose each other and lead to a minimum energy with a thermodynamically favored size for each gold-amine colloid system.

Regarding the mechanism of the digestive ripening process, it must proceed by an etching process where gold atoms or clusters are attacked by ligand molecules and lifted off the surface into solution, and then redeposited on other smaller particles. In this way, eventually a favored size is reached. This favored size is a minimum free energy of the whole system.

As for the interparticle separation, it seems that the bigger particles are more likely to aggregate into 3D superlattices, and the smaller ones are more likely to form separate particles. One possible explanation for this tendency can be rationalized from the attraction energy between the particles. To understand qualitatively the interparticle features, we have calculated the van der Waals attraction potential between the particle cores, utilizing the equation shown below [51]:

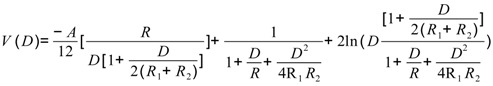


Here, A is the Hamaker constant, for gold it is 1.95 eV. R1 and R2 are the radii of the two spheres between which the attraction forces are to be calculated. D is the distance between the nearest surfaces of two adjacent spheres. Since the spheres are separated by the amine, we have assumed that the two alkyl chains attached to the two adjacent gold particles are fully interdigitated; that means D equals to the chain length of the amine. *R* is the reduced radius given by 2*R*_1_*R*_2_/(*R*_1_ + *R*_2_); in our case *R*_1_ = *R*_2_ = *R*. The attraction potentials turn out to be –2.7 eV, –1.5 eV, –0.61 eV, –0.72 eV and –0.62 eV for Au-C_4_N, Au-C_8_N, Au-C_12_N, Au-C_16_N and Au-C_18_N, respectively. From these results, we can clearly expect Au-C_4_N and Au-C_8_N to favor aggregation since the attraction energies are larger than the others.

### 3.2. Gold-Amine by the SMAD Method: The Effect of Alkyl Chain Length, Comparison between Inverse Micelle and SMAD Method

**Figure 14:**
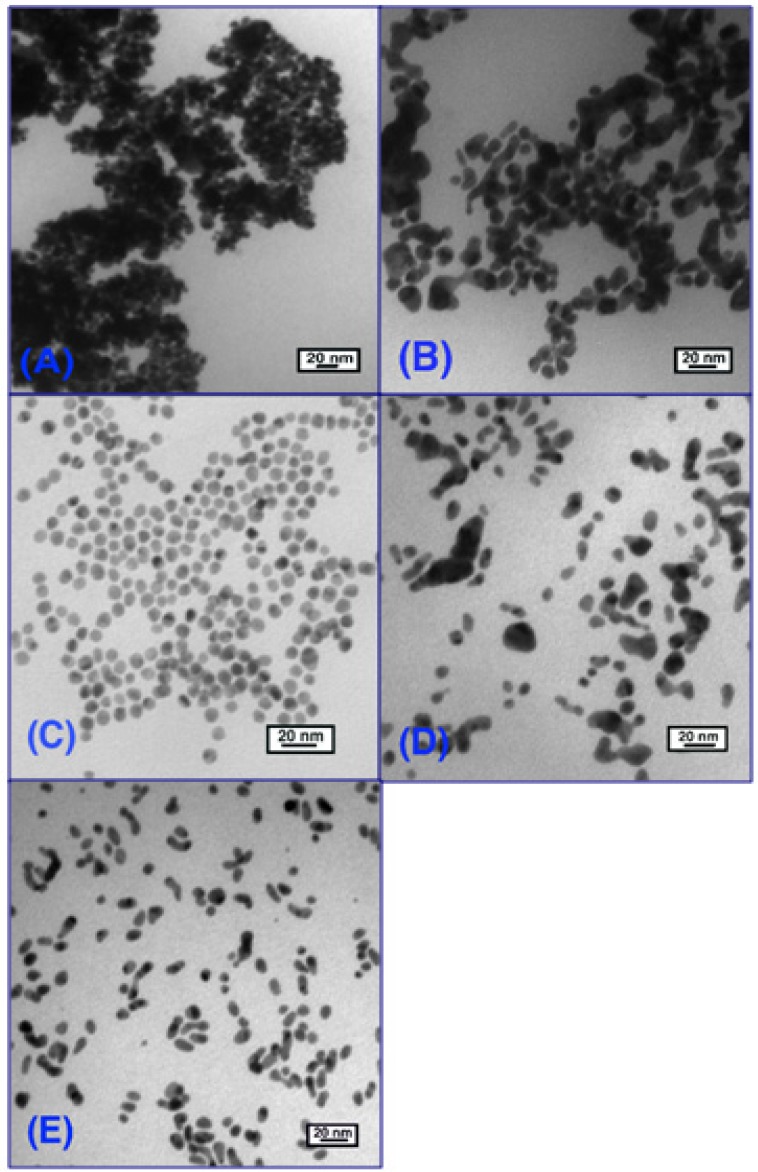
TEM images of (**A**) Au-C_4_N; (**B**) Au-C_8_N; (**C**) Au-C_12_N; (**D**) Au-C_16_N; and (**E**) Au-C_18_N colloids after digestive ripening in toluene.

**Figure 15:**
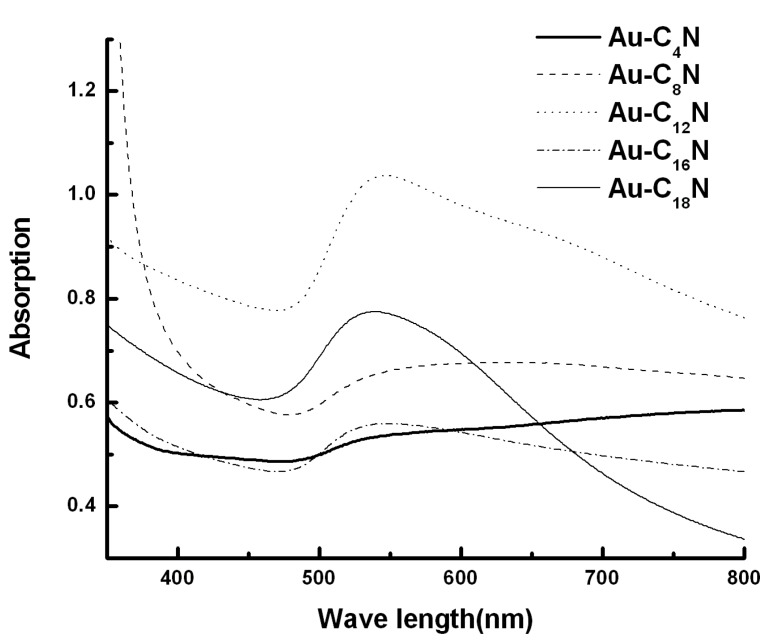
Comparison of surface plasmon resonance of these gold-amine colloids after digestive ripening in toluene.

Gold-amine colloids that were capped by C_4_N, C_8_N, C_12_N, C_16_N and C_18_N ligands were also synthesized by the SMAD method. The as prepared colloids which were pre-stabilized by butanone were divided into five portions along with different amine ligands. Then the solvent butanone was removed from those colloids by vacuum, and toluene was added. The colloids were re-dispersed and digestively ripened in toluene. TEM and UV-Vis were also conducted to characterize the morphologies and particle size distributions of the gold colloids before and after digestive ripening. However, it seems that there are no significant changes during the digestive ripening process. Before the digestive ripening, the color of the gold-amine colloids changed from dark blue to dark purple as the ligand length increases, and some precipitates formed at the bottom, while after being refluxed in toluene and cooled down to room temperature, the color of the each gold colloid stayed the same. The Au-C_4_N and Au-C_8_N colloids settled down easily with almost colorless supernatants. Some precipitates were also formed in Au-C_12_N, Au-C_16_N and the supernatant was purple color. Figure 14 shows the TEM images for Au-C_4_N, Au-C_8_N, Au-C_12_N, Au-C_16_N and Au-C_18_N colloids after digestive ripening. Very interesting results were obtained, as can be clearly seen. The morphology of these gold colloids, except for Au-C_12_N, were no longer polyhedral spheres; they tended to form rod shapes, and the Au-C_4_N and AuC_8_N particles aggregated together forming 3D superlattices, while the Au-C_16_N and Au-C_18_N colloids have a tendency to organize into 2D layers. As for Au-C_12_N, after digestive ripening the particles were organized nicely forming 2D monolayer spherical particles with mean size 9.4 ± 1.2 nm, compared with the Au-C_12_N colloid synthesized by inverse micelle method with particles mean size 8.8 ± 1.2. The morphologies are similar, but particles sizes are bigger. The UV-Vis spectra were in total agreement with those observations. As can be seen from Figure 15, which shows the surface plasmon absorption of Au-C_4_N, Au-C_8_N, Au-C_12_N, Au-C_16_N and Au-C_18_N colloids after digestive ripening, the plasmon band of both the Au-C_4_N and Au-C_8_N have a large tail above 700 nm, which illustrates that the particles are forming aggregates. Au-C_12_N colloids have a shoulder centered at 630 nm after the 530 nm peak, which reveals some particles forming 3D superlattices. As for Au-C_16_N and Au-C_18_N, we can see a broad gold plasmon peak around 530 nm.

The morphologies of these gold amine colloids synthesized by inverse micelle and SMAD are quite different. The morphologies of the inverse micelle colloids are more like polyhedral spheres, nicely arranged, while the shape of the SMAD colloids are very irregular except for Au-C_12_N. The particle morphologies of the Au-C_12_N colloids by both methods are very similar, but the SMAD method yields bigger particle sizes. The differences can be explained by the different synthesis route of the two techniques. In the inverse micelle method, the nucleation and crystal growth are controlled by three factors: (1) the presence of DDAB; (2) the controlled reaction temperature; and (3) the reaction speed. While in the SMAD method, atoms aggregate and crystals grow in a more chaotic manner, since the aggregation speed of the atoms is too fast to control. Previous work in our group has reported that particles synthesized by the inverse micelle technique preferentially assemble into face-centered cubic (fcc) structures, while the SMAD nanoparticles behave like “hard” spheres and predominantly organize into hexagonal close-packed (hcp) nanocrystal superlattices [44]. Although the digestive ripening process was carried out in both synthesis methods, it seems that it was less effective regarding core particle size adjustments when the SMAD method was used, as compared to the inverse micelle method.

Apparently, the SMAD method yields particles, initially, that are larger and perhaps more crystalline, and thus more resistant to the digestive ripening/etching process, when compared to initially formed particles by inverse micelle.

### 3.3. Comparison between Alkylthiol and Alkylamine as the Capping Ligand for Gold Colloids

The properties of thiol capped gold nanoparticles have been well investigated, including their monodispersity, the fact that they are easily isolated, as well as their nature to self-assemble into superlattices. The most stable gold colloid was that using dodecanethiol (C_12_H_25_SH) as the capping agent. After digestive ripening, it yielded a monodispersed gold colloid with an average size of 4.7 nm by inverse micelle method [52]. In contrast, digestively ripened by dodecylamine (C_12_N) leads to larger particles with an average size of 8.8 nm. Recently, the nature of the ligand-gold bonding has been reported that thiol is bound to the gold surface as a thiolate with the release the hydrogen [43]. Compared to sulfur in thiol ligands, nitrogen in amine ligands binds less strongly with the gold surface. Thus, we can conclude that less ligand-gold binding energy leads to the larger core-particle sizes, which is also in support of the proposal we discussed above, that a combination of a curvature-dependent surface energy and the ligand-gold binding energy leads to the particular size of nanoparticle.

### 3.4. Summary of Alkyl Amines

For the gold alkyl-amine colloids synthesized by the inverse micelle method, the digestive ripening process substantially changed the particle size distribution, and transformed the polydispersed colloid into a monodispersed colloid. As the chain length increases from butylamine (C_4_N) to octadecylamine (C_18_N), the gold particle size decreases with more narrowed size distribution, except for Au-C_12_N colloid, which yields the smallest particle sizes and the most narrow size distribution. The bigger particles, like Au-C_4_N and Au-C_8_N, seem to very easily aggregate and almost all the particles settled down and formed 3D superlattices. Smaller particles, like Au-C_12_N, Au-C_16_N and Au-C_18_N, have a tendency to form 2D monolayers; but when kept for a longer time, can still form 3D superlattices.

As for the gold colloids produced by the SMAD method, the digestive ripening process is not as effective as for the inverse micelle particles. TEM images show that the dodecylamine capped Au colloids (C_12_N) have an average particle size of 9.4 ± 1.2 nm, the particle sizes are slightly (9.4 *vs.* 8.8) bigger than the Au-C_12_N colloids by inverse micelle method. The morphologies of Au colloids that are capped by C_4_N, C_8_N, C_16_N and C_18_N are irregular, so it is hard to determine the particle size and size distribution. Au-C_4_N and Au-C_8_N very easily aggregate and settled down.

Compared with the alkyl-thiol as the capping ligand for gold colloids, amine capped gold nanoparticles are bigger, which indicates that stronger ligand binding can lead to smaller particle sizes. The final particle sizes of the colloid were determined by a combination of a curvature-dependent surface energy and the ligand-gold binding energy to reach a minimum free energy of the system.

### 3.5. Aromatic Amines (More Reactive than Alkyl Amines)

#### 3.5.1. Gold-Aniline Colloid in Butanone System

**Figure 16:**
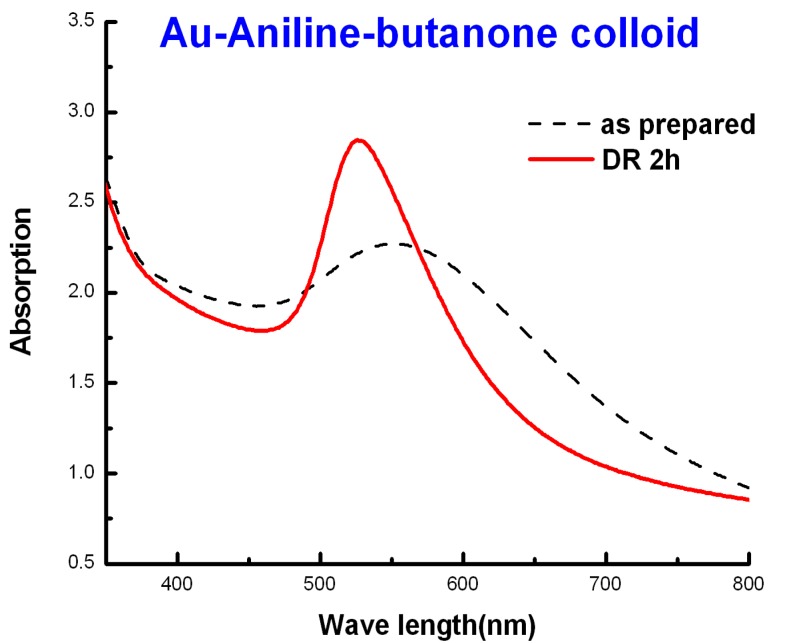
UV-visible spectrum of Au-aniline in butanone before and after digestive ripening, and the digestive ripening process kept going for 2 h.

The SMAD as prepared Au-aniline-butanone colloid has a dark purple color and widely variable particle sizes (10–150 nm). After the digestive ripening process, the color of the colloid changed from dark purple to bright purple. TEM studies show nearly spherical particles mostly in the 10–25 nm range. Thus, the digestive ripening procedure did improve the size distribution of the gold nanoparticles. This is also confirmed by the UV/Vis absorption spectrum (Figure 16). The surface plasmon resonance (SPR) peak of the as prepared colloid shows a broad peak with a low absorption maximum; but as the digestive ripening process progresses, the SPR peak becomes sharper and the absorption maximum increases.

#### 3.5.2. Gold-Aniline Colloid in Toluene System

**Figure 17:**
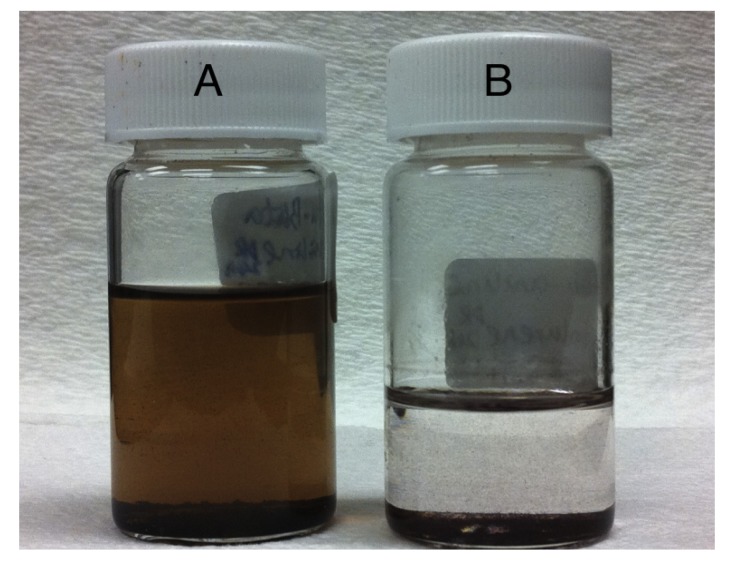
Photographs of (**A**) Au-aniline colloids in butanone seven days after digestive ripening; and (**B**) Au-aniline colloids in toluene seven days after digestive ripening.

**Figure 18:**
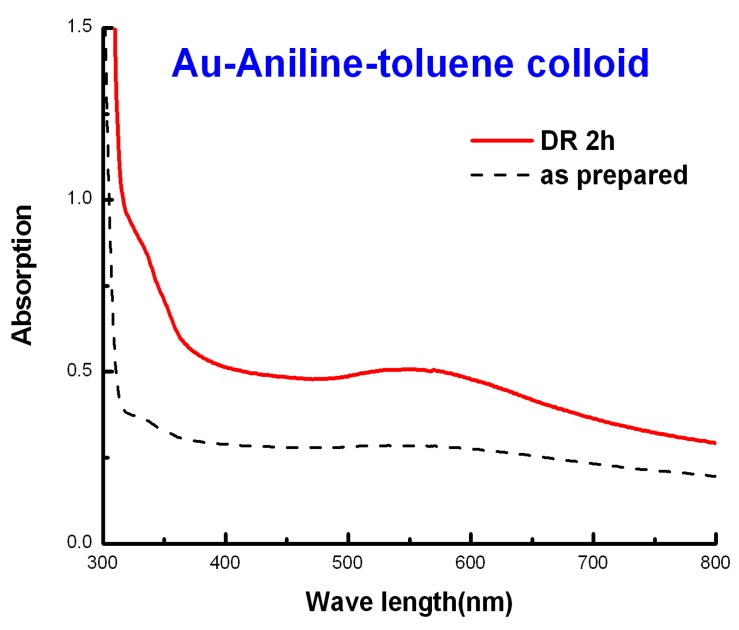
UV-Vis spectrum of Au-aniline colloids before and after digestive ripening 2 h in toluene.

In the gold-aniline-toluene system, gold nanoparticles precipitated during the warming up process, and after digestive ripening, big dark clusters formed at the bottom of the flask and the supernatant was light pink. Figure 17 shows photographs of Au-aniline colloids in both butanone and toluene digestive ripened after 7 days. As we can see, in the butanone, the gold colloid was yellow brown, and some gold particles precipitated. While in the Au-aniline-toluene system, the supernatant became nearly clear, almost all the particles precipitated to the bottom. The UV/Vis absorption spectrums (Figure 18) of the gold colloid before and after digestive ripening confirmed there was no significant change of the size distribution after digestive ripening. They are characterized by a broad plasmon absorption band with no definite maximum.

The results indicate that toluene is not a good condensing solvent, compared with butanone for the gold nanoparticles, and the digestive ripening process did not work as well in toluene. One possible explanation for the different stability of the two gold colloid systems is as follows: butanone is a polar solvent and with its nonbonding electron pairs it can act as a good ligand for gold nanoparticles at the preliminary stage. While the polarity of toluene is less, it is harder for toluene to bind with gold atoms, so gold atoms aggregated together and big clusters formed. Once the big gold clusters formed, it is even harder for gold nanoparticles to undergo the digestive ripening process in the presence of aniline.

We conclude that toluene is not as effective as butanone as a cocondensing solvent, but is effective as a digestive ripening solvent after the gold-butanone colloid is prepared and the crude particle isolated.

#### 3.5.3. Gold-Pure Aniline Colloids

**Figure 19:**
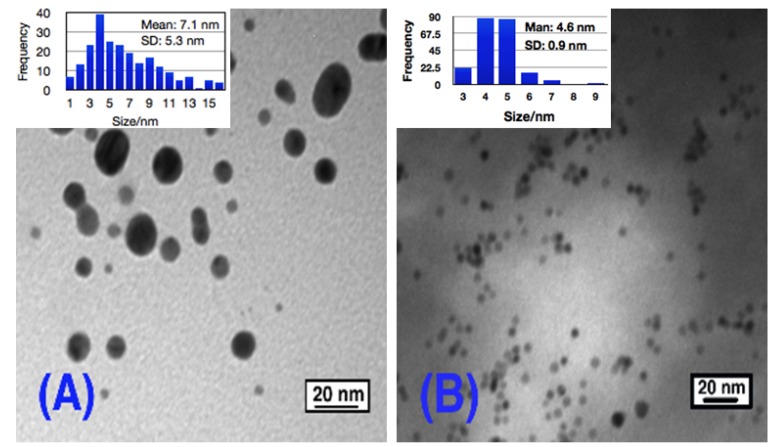
TEM images of (**A**) Au-pure aniline colloids after 7 days; (**B**) Au-pure aniline colloids after digestive ripening. The particle size distributions of the Au-aniline colloid are given in the inset of (**A**) and (**B**), the mean diameter of the particle size is 7.1 ± 5.3 nm, 4.6 ± 0.9 nm respectively.

**Figure 20:**
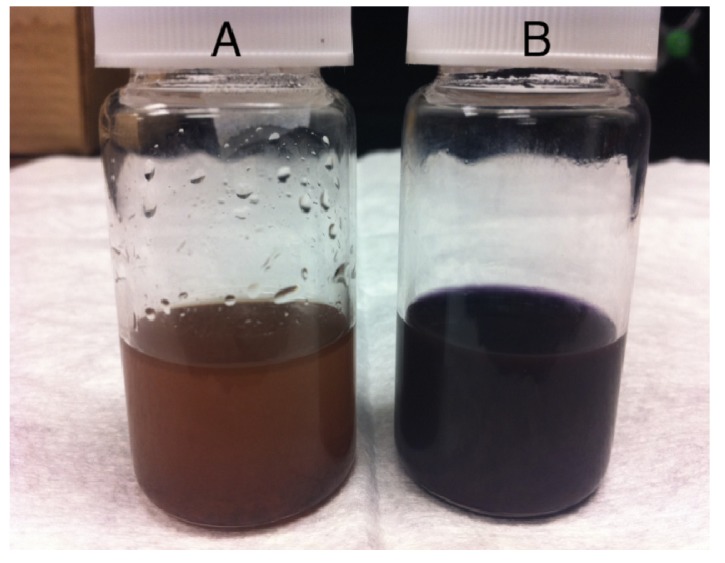
Photographs of (**A**) Au-pure aniline colloids after digestive ripening 2 h; and (**B**) the Au-pure aniline colloids that was ripened for 7 days.

In the gold-pure aniline system, the gold colloids were under reflux in pure aniline. TEM studies (Figure 19B) show that the average particle size is 4.6 ± 0.9 nm, compared with the gold-aniline in butanone system, in which the mean particle size is 28 ± 20 nm, so both the particle sizes and size distribution are much smaller. Figure 19A shows the TEM of the same gold-pure aniline colloids after 7 days. As we can see the particles grew and aggregated to bigger ones, with mean particle size of 7.1 ± 5.3 nm. Figure 20A shows the photographs of Au-pure aniline colloids after 2 h digestive ripening. The color of the gold colloid was brownish yellow, and colloid was uniform and quite stable. When kept in process for a longer time, the colloid became darker but still homogenous without any precipitates. Figure 20B shows the digestively ripened gold-pure aniline colloid after one week.

From the results above, we could draw a conclusion that aniline does play a significant role as a capping ligand in stabilizing the gold colloid, and as the concentration of the aniline goes higher the gold colloid is more stable and the particle sizes, as well as the size distribution, become smaller. The color of the colloid became darker when kept for a longer time, probably due to the gold particles rearranging, but particle sizes are still quite small since there were no precipitates formed in this system.

#### 3.5.4. Gold-Phenethylamine Colloids

**Figure 21:**
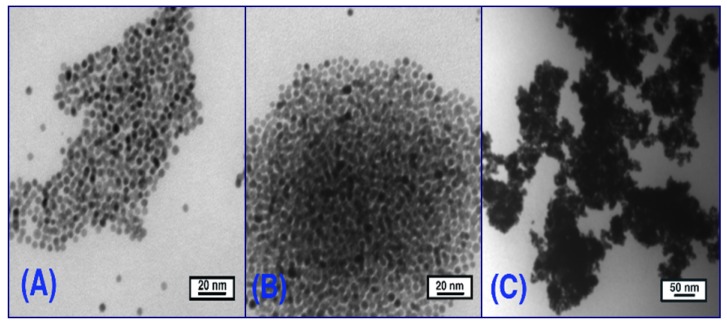
TEM images of (**A**) As prepared Au-phenethylamine colloids in butanone; (**B**) Au-phenethylamine colloids after 2 h digestive ripening in butanone; and (**C**) Au-phenethylamine after 3 h digestive ripening.

**Figure 22:**
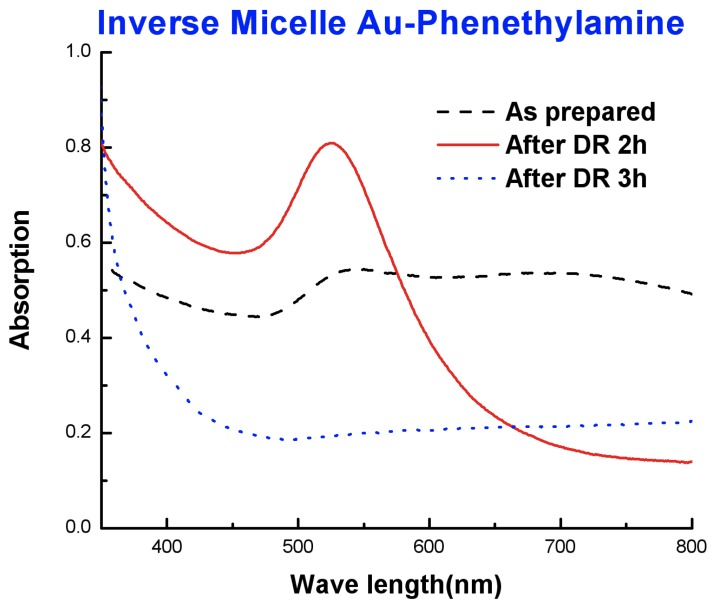
UV-visible spectrum of Au-phenethylamine colloids before and after digestive ripening.

Phenethylamine is a commonly used aromatic amine, which has similar structure as aniline. The gold-phenethylamine colloids were first synthesized by the inverse micelle method. The digestive ripening process was carried out in butanone solution, and some interesting results were revealed. Figure 21, Figure 22 show the TEM and UV-Vis spectra results. As can be clearly seen, the gold-phenethylamine (Figure 21) particles arranged nicely and tended to form a 2D monolayer. As digestive ripening progressed, the particles began to aggregate together (Figure 21), and after 3 h almost all of the particles settled down and aggregated together, as shown in Figure 21C. The UV-Vis spectra also confirm this, as it shows in Figure 22. After digestive ripening 2 h, the plasmon band became sharp and narrowed, indicating that the digestive ripening works during the first 2 h, when polydispersed colloids are transformed into monodispersed one. However, after 3 h, all of the particles aggregated and precipitated, as confirmed by UV-Vis spectra showing a broad band with no definite peak.

The SMAD method, using butanone as the cocondensing solvent, also successfully produced small, fairly monodisperse particles that showed a strong tendency to aggregate into superlattices.

#### 3.5.5. Summary of Aromatic Amines

Aniline capped gold colloids were successfully synthesized in three different systems by the SMAD method. Butanone turned out to be a better choice for the condensing solvent as a preliminary capping ligand compared with toluene. Digestive ripening took place and greatly improved the size distribution of the gold-aniline-butanone system. The gold-aniline colloid in pure aniline yields much smaller and more stable gold nanoparticles, which indicates that ligand concentration efficiently affects the particle sizes.

It was found that these aromatic amines worked well as digestive ripening ligands, but the relatively short carbon ligand (C_6_H_5_– or C_6_H_5_CH_2_CH_2_–) gave the particles poorer solubility and therefore a tendency to aggregate and settle out of solution.

## 4. Conclusions

From this broad study, the following conclusions can be drawn:

(1) Amines are useful capping ligands for gold nanoparticles, and stabilize as Au-NH_2_R bonds without loss of hydrogen;
(2) Amines and thiols are good digestive ripening solvents, although thiols are the better of the two;
(3) With inverse micelle produced particles, all amines behave as good digestive ripening agents;
(4) With SMAD produced particles, only selected amines (C_12_H_25_NH_2_, C_6_H_5_–NH_2_, C_6_H_5_CH_2_CH_2_NH_2_) serve to digestively ripen the particles, while other alkyl amines were less favorable. Reactivity and chain length are both important;
(5) Inverse micelle prepared particles are more reactive and susceptible to digestive ripening than SMAD prepared.
